# Cell Adhesion in Cancer

**DOI:** 10.1155/2012/965618

**Published:** 2012-03-20

**Authors:** Eok-Soo Oh, Motoharu Seiki, Martin Gotte, Jun Chung

**Affiliations:** ^1^Department of Life Sciences, Division of Life and Pharmaceutical Sciences and the Center for Cell Signaling & Drug Discovery Research, Ewha Womans University, Seoul 120-750, Republic of Korea; ^2^Division of Cancer Cell Research, Institute of Medical Science, University of Tokyo, Minato-ku, Tokyo 108-8639, Japan; ^3^Department of Gynecology and Obstetrics, Medical Center, University of Münster, D-48149 Münster, Germany; ^4^Department of Biochemistry and Molecular Biology, Louisiana State University Health Sciences Center, 1501 Kings Highway, Shreveport, LA 71103, USA

During cancer progression, cells lose their original tissue contacts, move through the extracellular matrix (ECM), enter into the lymphatic and/or blood system, extravasate, and ultimately form new tumors. Therefore, tumor cells inevitably experience alterations in cell-cell and cell-ECM adhesion and the transformation activities of tumor cells are highly influenced by cell adhesion via adhesion receptors such as cadherins, integrins, cell surface proteoglycans, and tetraspanins. These adhesion receptors, together with extracellular ligands in the tumor microenvironment, couple the extracellular environment to intracellular signals, thereby enhancing cancer cell migration, invasion, proliferation, and survival. Therefore, knowledge of the role of cell adhesion in cancer is key to understanding the development of cancer, and such knowledge could potentially form the basis for effective approaches to cancer treatment. Therefore, in this issue, we have invited several authors to address such issues.

 The special issue will be composed of three parts. A set of four papers discuss regulatory mechanisms in the light of interesting issues that have arisen recently, such as the role of cell-ECM interactions in genetic mutations in tumors, as well as the important roles of lipid rafts, turnover of focal adhesions, and anoikis resistance in cancer cell adhesion and migration. All of these characteristics have been shown to be important in cancer cell adhesion, and recent findings provide potential mechanisms of tumor cell interactions and metastatic activities.

Another set of five papers of this special issue address the molecular level regulatory role of cell adhesion molecules such as integrins and the immunoglobulin super family and adhesion-related regulatory molecules such as plakoglobin, HIC-5, and prostaglandins. All of these molecules have received considerable attention in cell adhesion research and have distinct role(s) in different aspects of cancer progression, but all are important regulators of human carcinogenesis through their capacity to regulate cancer cell adhesion. However, because their roles in cancer cell adhesion and metastasis are still under investigation, this special issue addresses their functional significance, particularly in the context of cancer cell adhesion.

A set of two papers address the possibility of suppressing tumors by regulating cell adhesion status using ECM-derived functional peptides or a physiological regulator such as heparin. Research in this area will improve the understanding of cancer progression and may provide the basis for a new strategy for treating cancer.

*Eok-Soo Oh* *Eok-Soo Oh* *Motoharu Seiki* *Motoharu Seiki* *Martin Gotte* *Martin Gotte* *Jun Chung* *Jun Chung*

